# Biomedical research; what gets funded where?

**DOI:** 10.2471/BLT.19.240499

**Published:** 2019-08-01

**Authors:** Taghreed Adam, Ambinintsoa H Ralaidovy, Soumya Swaminathan

**Affiliations:** a Science division, World Health Organization, avenue Appia 20, 1211 Geneva 27, Switzerland.

The World Health Organization’s (WHO) Global Observatory on Health Research and Development was established in January 2017, following a request from the World Health Assembly to assist partners in coordinating and prioritizing new investments in health research and development, particularly in areas where the incentive for investment is insufficient.^1^ These areas typically include poverty-related diseases or rare diseases, where no market exists to sufficiently offset the cost of product development.

The observatory has since expanded its scope and mandate to cover all diseases and all types of health research in response to WHO’s *Thirteenth General Programme of Work*.^2^

In July 2019, the observatory published for the first time a comprehensive overview of health products for all indications (medicines, vaccines and diagnostics that include an active pharmaceutical ingredient), from discovery to market launch, using the AdisInsight database.^3^^,^^4^ The analysis of more than 86 000 products developed since 1995 shows that of the 14 999 products that are currently in a clinical phase of development, 87% (13 004) are for noncommunicable diseases and 9% (1319) for communicable, maternal, perinatal and nutritional conditions. Around 48% (6221) of products for noncommunicable diseases are for malignant neoplasms and around 80% (1047) of products for communicable, maternal, perinatal and nutritional conditions are for infectious and parasitic diseases.^4^ Less than 0.5% of active products (168 out of 35 770) target a disease on the WHO list of neglected tropical diseases, and around 0.4% (152) of active products are targeting a pathogen on the WHO list of research and development blueprint priority pathogens.^5^^,^^6^

These findings and other recent analysis of the observatory’s data show little indication that decisions on new investments on health research and development are evidence-informed or prioritized, or that they reflect the public health needs of people living in low- and middle-income countries.

For example, the large number of products for noncommunicable diseases is welcome, given that these diseases are the leading cause of death (63% of global deaths, that is, more than 36 million people annually). However, there is little evidence that the rapid epidemiological transition and rising burden in low- and middle-income countries are being considered. As low- and middle-income countries already bear 86% of the burden of premature deaths from noncommunicable diseases,^7^ their needs should be reflected in the distribution of research grants and clinical trials across the world. An analysis of the distribution of grants for biomedical research by 10 major funders of health research, using data in the World RePORT platform, showed that globally, almost three-quarters of all grants in 2016 were for noncommunicable diseases (72%; 40 035), followed by communicable, maternal, perinatal and nutritional conditions (20%; 11 123) and injuries (6%; 3056). Yet when these data are analysed by lower-middle and low-income country status, respectively, the findings are reversed: in these countries, 71% and 85% of grants were for communicable, maternal, perinatal, and nutritional conditions.^8^^,^^9^

Responding more effectively and equitably to the health-care needs of people with such diseases, and influencing public policies in sectors outside health that tackle shared risk factors, such as tobacco use, unhealthy diet and physical inactivity, could prevent most premature deaths from noncommunicable diseases. The needs of low- and middle-income countries, either in implementation or adapted health products and technologies, all require proactive prioritization of research and development activities. Addressing these needs through locally relevant research questions would contribute to halting this increasing burden of largely preventable diseases.^7^

WHO’s general programme of work elaborates a set of priorities based on public health needs, emphasizing the importance of working with partners and stakeholders. The programme also highlights the importance of coordinating health research and development activities and the role of evidence-informed decision-making to ensure the most efficient and equitable use of resources.^2^

The observatory aims to assist with the call for coordinated efforts by providing decision-makers around the world with access to a comprehensive set of analyses on who is doing what in health research and product development, where and with what resources. Expanding data sharing of this type of information and using the data better to coordinate new investments in health research and development, including implementation and health systems research, is crucial. Doing so will ensure that when technologies are available and effective, they are implemented efficiently and equitably; and when they are not, decisions to prioritize new investments on health research and development are coordinated and oriented towards public health needs.

## References

[1] Global observatory on health R&D [internet]. Geneva: World Health Organization; 2019. Available from: www.who.int/research-observatory/en/ [cited 2019 Jun 6].

[2] Thirteenth general programme of work 2019–2023. Geneva: World Health Organization; 2019. Available from: https://apps.who.int/iris/bitstream/handle/10665/324775/WHO-PRP-18.1-eng.pdf [cited 2019 Jul 9].

[3] AdisInsight [database]. Geneva: Springer Nature Switzerland AG; 2019. Available from: https://adisinsight.springer.com/ [cited 2019 Jun 25].

[4] Health products in the pipeline from discovery to market launch for all diseases. Geneva: World Health Organization; 2019. Available from: https://www.who.int/research-observatory/monitoring/processes/health_products/en/ [cited 2019 Jun 25].

[5] Neglected tropical diseases. Geneva: World Health Organization; 2019. Available from: https://www.who.int/neglected_diseases/diseases/en/ [cited 2019 Jul 9].

[6] List of Blueprint priority diseases. Geneva: World Health Organization; 2018. Available from: https://www.who.int/blueprint/priority-diseases/en/ [cited 2019 Jun 25].

[7] Global action plan for the prevention and control of noncommunicable diseases 2013–2020. Geneva: World Health Organization; 2013. Available from: https://www.who.int/nmh/events/ncd_action_plan/en/ [cited 2019 Jun 25].

[8] Number of grants for biomedical research by funder, type of grant, duration and recipients (World RePORT). Geneva: World Health Organization; 2019. Available from: https://www.who.int/research-observatory/monitoring/inputs/world_report/en/ [cited 2019 Jun 25].

[9] World RePORT. Bethesda: National Institutes of Health; 2018. Available from: https://worldreport.nih.gov/ [cited 2019 Jun 25].

